# Genomic signatures of protease and reverse transcriptase genes from HIV-1 subtype C isolated from first-line ART patients in India

**DOI:** 10.6026/97320630018371

**Published:** 2022-04-30

**Authors:** Sushanta Kumar Barik, Keshar Kunja Mohanty, Shripad A Patil, Srikanth Prasad Tripathy, Dharmendra Singh, Luke Eilzabeth Hanna, Ramesh Karunaianantham, Sathyamurthi Pattabiraman, Tej Pal Singh, Rekha Tandon, Srikanta Jena

**Affiliations:** 1 ICMR-National JALMA Institute for Leprosy and Other Mycobacterial Diseases, Agra, Uttar-Pradesh, India; 2 ICMR-National Institute for Research in Tuberculosis, Chetpet, Chennai, Tamil Nadu, India; 3 Sarojini Naidu Medical College and Hospital, Agra, Uttar-Pradesh, India; 4 Ravenshaw University, Odisha, Cuttack, India

**Keywords:** Archived DNA, Signature, Protease (PR), Reverse transcriptase (RT), HIV-1

## Abstract

Genomic signatures of the protease and reverse transcriptase gene of HIV-1 from HIV infected North Indian patients who were under ART from 1 to ≤ 7 years were analyzed. The DNA from plasma samples of 9 patients and RNA from 57 patients were isolated and subjected to amplification for the protease and reverse transcriptase gene of HIV-1 subtype C. Then sequencing was carried out following the WHO dried blood spot protocol. The drug resistance mutation patterns were analyzed using the HIV Drug Resistance Database, Stanford University, USA. Lamivudine-associated drug-resistance mutations such as M184V/M184I, nevirapine-associated drug resistance mutations Y181C and H221Y, and efavirenz-associated drug resistance mutations M230I were observed in reverse transcriptase gene of archived DNA of two HIV-1 infected patients. No mutation was observed in the remaining 7 patients. Various computational tools and websites like viral epidemiological signature pattern analysis (VESPA), hyper mutation, SNAP version 2.1.1, and entropy were utilized for the analysis of the signature pattern of amino acids, hyper mutation, selection pressure, and Shannon entropy in the protease and reverse transcriptase gene sequences of the 9 archived DNA, 56 protease gene and 51 reverse transcriptase gene from the HIV-1 DNA amplified sequences of RNA. The HIV-1 Subtype-C (Gene bank accession number: AB023804) and first isolate HXB2 (Gene bank accession number: K03455.1) was taken as reference sequence. The signature amino acid sequences were identified in the protease and reverse transcriptase gene, no hyper mutation, highest entropy was marked in the amino acid positions and synonymous to non-synonymous nucleotide ratio was calculated in the protease and reverse transcriptase gene of 9 archived DNA sequences, 56 protease and 51 reverse transcriptase gene sequences of HIV-1 Subtype C isolates.

## Background:

Acquired immunodeficiency syndrome (AIDS) is mainly caused by human immunodeficiency virus-1 (HIV-1) and human-immunodeficiency virus-2 (HIV-2). The molecular characterization of the human immunodeficiency virus-1 and human immunodeficiency virus-2 in Yaounde, Cameroon was reported [1]. The length of the HIV-1 genome is approximately 9.1 kilobases. The genome consists of 15 proteins that completely regulate the life cycle of viruses within human beings [2]. HIV-1 replicates within the host [3]. The reverse transcriptase gene of the HIV-1 synthesized the DNA strands and the protease gene cleaved the strands to form the mature particles [4].The reverse transcriptase and protease gene of HIV-1 were sequenced for drug resistance studies [5]. Several types of antiretroviral therapy are used to treat the HIV-1 patients in the antiretroviral therapy program in India. According to guidelines, the HIV-1 patients initially started the first line antiretroviral therapy which consists of nucleoside reverse transcriptase inhibitors (NRTIs) like zidovudine, lamivudine, tenofovir, abacavir and non-nucleoside reverse transcriptase inhibitors (NNRTIs) like efavirenz and nevirapine etc. (ART guidelines) [6,7]. If the patients were on the failure of first line ART, the switch over to second line antiretroviral therapy is an option to decrease the viral copy number [8]. High viral load or low CD4 count as well as no drug resistance mutations was associated with mortality in AIDS defining patients [9]. However, the drug resistance mutation analysis is essential in patients infected with HIV-1 [10]. The list of pattern of mutations with the drug panels were highlighted by the expert groups [11]. The full-length of the HIV-1 proviral genome was characterized undergoing the firstline highly active antiretroviral therapy [12]. The HIV-1 proviral DNA drug resistance mutations were reported in a community treatment program [13]. Drug resistance mutation analysis of the archived DNA could help in choosing the proper regimen at a low level or suppressed viremia patients [14]. The functional analysis of the genomic signatures of HIV-1 gives important information on strain subtypes, epidemiological signatures, nucleotide substitution rates, Shannon entropy etc. Molecular epidemiology of human immunodeficiency virus transmission was reported in a dentist with AIDS [15]. The study of signature pattern analysis was analysed through the viral epidemiological signature pattern analysis (VESPA) [16]. The epidemiological signature pattern analysis of HIV-1 genome was reported in a Southern Indian clinical cohort study [17]. Therefore, it is of interest to document the genomic signatures of Protease and Reverse transcriptase gene of HIV-1 Subtype C isolated from the first line ART patients from India.

## Materials and Methods:

### Epidemiological Investigation of HIV-1 patients:

The patients were enrolled for first line ART at the antiretroviral therapy centre, Sarojini Naidu Medical College, Agra, India from December 2009 to November 2016 as per the treatment guidelines directed by National AIDS Control Organization (NACO), Govt. of India. The details of clinical and sociodemographic profile were collected as per the published leaflet [18]. These patients were on first-line ART such as ZLE (Zidovudine + Lamivudine + Efavirenz), ZLN (Zidovudine + Lamivudine + Nevirapine), TLE (Tenofovir + Lamivudine + Efavirenz), TLN (Tenofovir + Lamivudine + Nevirapine), SLE (Stavudine + Lamivudine + Efavirenz) and SLN (Stavudine + Lamivudine + Nevirapine). The details of the study of the genotyping including the polymerase chain reaction and sequencing primers as well as amplification conditions of two-step polymerase chain reaction (1st round and 2nd round) was reported earlier [19]. After genotyping, Drug resistance mutation analysis of archived DNA and plasma RNA samples were performed by the HIV drug resistance database, Stanford University, USA (http://hivdb.stanford. edu/pages/algs/sierra_sequence.html). The 7-protease gene and 9 reverse transcriptase genes of the archived DNA samples, 56 protease genes and 51 reverse transcriptase genes of RNA samples were considered through the genomic signatures in a molecular epidemiological study.

### Gene Bank accession number:

All the 57 partial polymerase gene sequences from RNA samples bearing the accession number MG788697 to MG788753 and the 9 partial polymerase gene archived DNA bearing the accession number MH503757 to MH503765 were available at NCBI, USA.

### A computational approach for the analysis of the archived DNA and plasma RNA:

The nucleotide sequences of the protease (PR) and reverse transcriptase (RT) gene were aligned using multiple sequence alignment tools (www.ebi.ac.uk/tools/mas). The nucleotide sequences were converted into amino acid sequences by EMBOSS Tran seq (www.ebi.ac. uk/ Tools/st/ emboss_transeq). The complete 9 PR gene lengths (nucleotide positions: 1 - 297) and the 7 RT gene lengths (nucleotide positions:310 - 988) of the archived DNA were taken for signature pattern analysis, hyper mutation analysis, selection pressure analysis, and Shannon entropy calculation. The complete 56 PR gene length (nucleotide positions: 1 to 297) and the 51 RT gene length (nucleotide positions: 310 to 988) were taken as the background sequences for signature pattern analysis, hyper mutation analysis, selection pressure analysis, and Shannon entropy calculation.

VESPA (Viral Epidemiology Signature Pattern Analysis) program was used for the comparison of amino acid sequences in the PR and RT genes for the archived DNA.VESPA program is available in the HIV databases (https://www.hiv.lanl.gov). The reference sequences of protease and reverse transcriptase genes of HIV-1 is being taken from the Gene Bank (accession number: AB023804) and HXB2 (accession number: K03455.1). The drug resistance-associated NRTI and NNRTI mutations were identified in the RT gene of 9 patients and the PR gene of 7 patients’ archived DNA sequences and then these sequences were taken as background sequences for VESPA, selection pressure, hyper mutation, and Shannon entropy analysis. The computational analysis of the sequences of the PR and RT gene of HIV-1 subtype C were performed by using the Los Alamos Laboratory, Pathogen Research Databases.(https://www.lanl.gov/collaboration/ pathogen-database). The VESPA analysis was performed (https://www.hiv.lanl.gov/content/sequence/VESPA/ vespa.html). The selection pressure analysis of the PR and RT gene was performed using the SNAP version 2.1.1 (synonymous non-synonymous analysis program tool (https://www.hiv. lanl.gov/content/sequence/SNAP/SNAP.html). The hyper mutation analysis of PR and RT gene of the archived DNA was performed. (www.hiv.lanl.gov/HYPERMUT/hypermut). Shannon entropy at each amino acid position of the PR and RT gene of archived DNA was calculated using the Shannon Entropy online tool at the Pathogen Research Data Bases (https://www.hiv.lanl.gov/content/sequence/ENTROPY/ entropy _option.html).

## Result and Discussion:

### Genomic signatures of the PR and RT gene of the archived DNA:

The details of the regimen profiles, viral load, and drug resistance mutations of protease and reverse transcriptase genes of the archived DNA isolated from 9 patients treated with first line ART are given in Table 1(see PDF). Drug resistance mutations M184V and M184I (lamivudine), Y181C and H221Y (nevirapine and efavirenz), and M230I (efavirenz) in the HIV-1 subtype C reverse transcriptase gene of the archived DNA were observed in two patients. No mutations were detected in the reverse transcriptase and protease gene in the remaining seven archived DNA. The VESPA software was used for amino acid comparison of the 9 archived DNA sequences in HIV-1 Subtype C along with the reference sequences of Mochizuki et al., 1999 (Gene bank accession number: AB023804) [20]. This reference sequence was an HIV-1 subtype C isolated from India in the year 1999. To check the atypical amino acid sequences in the archived DNA, the reference sequence (AB023804) was taken for the signature pattern study. Full length i:e 99 amino acids for the PR gene and 226 amino acids for the RT gene were analysed using VESPA. Signature amino acids viz., 36I, 82V were observed in the PR gene of the archived DNA. Signature amino acids 32T (aa position in RT:135T), 39A (aa position in RT :162A), 62T (aa position in RT :165T), 63K (aa position in RT:166K), 108R (aa position in RT:211R), and 214A (aa position in RT: 317A) were observed in the RT gene of the archived DNA. The signature amino acids of the PR and RT gene of reference sequence AB023804 with back ground sequences are given in Table 2(see PDF). The signature amino acids of the protease genes were found with the reference to background sequences at frequencies of 0.57 to 0.85. The signature amino acids of the reverse transcriptase genes were found with reference to background sequences at frequencies of 0.44 to 1.0. The signature frequency of protease and reverse transcriptase genes are given in Table 3 and Table 4(see PDF).

The selection pressure acting on the PR gene and the RT gene of archived DNA was evaluated at the coding level by subjecting the all-PR gene, RT gene sequences of clinical isolates along with the Indian Subtype C reference (AB023804). The ds/dn ratio of the PR genes (ds =0.08, dn =0.02, ds/dn =5.02) and RT genes were (ds =0.16, dn =0.02, ds/dn =6.31) calculated. Hypermutated proviral sequences were analysed through the identification of an excessive G→ A change pattern consistent with the APOBEC3G/F signature. The 9 RT and 7 PR archived DNA sequences were compared to a reference sequence, with G to A hypermutation. Nine patients had no G to A potential mutations in the PR and RT genes of HIV-1. Shannon entropy has been defined in terms of the probabilities of different sequences or clusters of sequences that can present at a given time point. To evaluate the genomic stability of protease and reverse transcriptase genes of archived DNA, the Shannon entropy analysis of each amino acid codon was performed. The 63 positions of amino acid P were observed as the highest random entropy =1.352 in the protease gene of the archived DNA. The amino acid T, A at positions 32 and 59, was observed as the highest random entropy=1.369 in the reverse transcriptase gene of the archived DNA. The entropy differences between the two sets were observed in ≤ 5 of the 100 randomizations. To check the atypical amino acid sequences in the archived DNA, the reference sequence (HXB2) was taken for the signature pattern study. Full length i:e 99 amino acids for the 7 PR genes and 227 amino acids for the 9 RT genes were analysed using VESPA. Signature amino acids viz., 3V, 12T, 14K,15I, 19L, 36M, 37S, 41R, 63L, 69H, 89L, 93I were observed in the 7 PR genes of archived DNA. Signature amino acids such as 18D, 32I, 59S, 70K, 74D, 97T, 104Q, 111L, 142V, 183T, 188E, 189V, 190I, 214V, 227Q were observed in the 9 RT genes of archived DNA. The signature amino acids of the protease gene were found with the reference to background sequences at frequencies of 0.57 to 1.00. The signature amino acids of the reverse transcriptase gene were found with reference to background sequences at frequencies of 0.44 to 1.00. The selection pressure acting on the 7 PR genes and the 9RT genes of th archived DNA was evaluated at the coding level by subjecting the all-PR gene, RT gene sequences of all clinical isolates along with the HXB2. The ds/dn ratio of the PR gene (ds =0.04, dn =0.06, ds/dn =0.61) and RT gene were (ds =0.03, dn =0.04, ds/dn =0.63) calculated. Hypermutated proviral sequences were analysed through the identification of an excessive G→A change pattern consistent with the APOBEC3G/F signature. The 9 RT and 7 PR archived DNA sequences were compared to a reference sequence, with G to A hypermutation. Seven patients and eight patients had no G to A potential mutations in the PR and RT genes of HIV-1 subsequently but one patient developed G to A hypermutation (Fisher extract P Value= 0.0001) in the RT gene of HIV-1 when compared with HXB2. Shannon entropy has been defined in terms of the probabilities of different sequences or clusters of sequences that can present at a given time point. To evaluate the genomic stability of protease and reverse transcriptase genes of the archived DNA, the Shannon entropy analysis of each amino acid codon was performed. The 9th positions of amino acid P were observed as the highest random entropy =1.074 in the protease gene of archived DNA. The amino acid S and A at positions 59th and 194th was observed as the highest random entropy =1.273 and 1.149 in the reverse transcriptase gene of archived DNA. The entropy differences between the two sets were observed in ≤ 5 of the 100 randomizations. The signatures and signature frequency of 7 PR and 9RT genes were given in Table 5, Table 6, Table 7 and Table 8(see PDF).

### Genomic signatures of the protease and reverse transcriptase gene of the plasma RNA:

Viral epidemiological signature pattern analysis (VESPA) was completed with 56-protease gene encoded amino acid sequences of HIV-1 subtype C along with the reference sequence of HIV-1 subtype C (Gene bank accession number: AB023804). In this VESPA analysis, 56-amino acid sequences of full-length PR gene taken as back ground sequences and the accession number: AB023804 taken as a query sequence (Reference sequence) in the analysis tool. The signature amino acids viz., 14K, 19T, 36I, 82V were observed in the 56-PR gene sequences of HIV-1 subtype C where as 14R, 19I, 36V, 82I were observed in the query sequence of HIV-1 subtype C. Then again, we compared the 56- amino acid sequences of HIV-1 subtype C with the query sequence of HXB2 (accession number: K03455) [21]. The signature amino acids viz., 3I,12S,15V,19T,36I,37N,41K,63P,69K,89M,93L were observed in the 56-PR gene sequences of HIV-1 subtype C where as 3V, 12T, 15I, 19L, 36M, 37S, 41R, 63L, 69H, 89L, 93I were observed in the PR gene sequence of HXB2. The details of the signature pattern and frequency analysis are given in tables: The VESPA analysis of the 56-PR gene sequences along with the accession number: AB023804 is given below in Table 9(see PDF).

VESPA analysis of the 51 RT gene sequences (1 to 320 amino acids) of HIV-1 subtype C was compared with the query sequence (reference sequence) of HIV-1 subtype C (Genbank accession number: AB023804). In this comparison, the signature amino acids such as 36E, 69T, 103N, 165T, 166K, 184V, 286T in the 51 RT gene of HIV-1 subtype C but the signature amino acids 36A, 69I, 103K, 165I, 166R, 184M, 286A were observed in the reference sequence accession number: AB023804. In another comparison, the 51-RT gene sequences of HIV-1 subtype C were compared with the reference sequence of HXB2 (accession number: K03455). In this comparison study, the signature amino acids such as 35T, 39D, 48T, 60I, 103N, 121Y, 173A, 177E, 184V, 200A, 207E, 211K, 214F, 245Q, 291D, 292I, 293V were observed in the 51 RT gene sequences of HIV-1 subtype C. The signature amino acids such as 35V, 39T, 48S, 60V, 103K, 121D, 173K, 177D, 184M, 200T, 207Q, 211R, 214L, 245V, 291E, 292V, 293I.

### Shannon entropy calculation of the protease an→d reverse transcriptase gene from plasma RNA:

To evaluate the genomic stability of protease and reverse transcriptase gene of the HIV-1 subtype C, the Shannon entropy analysis of each amino acid codon was performed with the reference HXB2 (accession number : K03455) and Indian subtype C (AB023804). The amino acid positions A121= 1.151, I135= 1.338, H162= 1.136 were observed as the highest random entropy in the reverse transcriptase gene of HIV-1 subtype C with the reference sequences (AB023804) and HXB2. Similarly, the amino acid positions S12=0.579, X19=0.830, I36=0.546, N37=0.722 were observed as the highest random entropy in the protease gene of HIV-1 subtype C with the reference sequences (AB023804) and HXB2. The entropy differences between two sets were observed in 100 randomizations with replacement, cut off for conserved signature = 5.

### Evaluation of selection pressure:

The selection pressure acting on the PR gene and RT gene of DNA was evaluated at codon level by subjecting all 56 PR gene and 51 RT gene sequences of clinical isolates along with the Indian subtype C reference (AB023804) and HXB2. The ds/dn ratio of the PR gene and RT gene with reference sequence AB023804 (ds = 0.113, dn =0.026, ds/dn =4.722 and (ds =0.135, dn=0.024, ds/dn = 5.992) were calculated. The ds/dn ratio of the PR and RT gene with reference sequence HXB2 (ds= 0.405,dn=0.059, ds/dn=7.075) and (ds=0.497,dn=0.040,ds/dn=12.758) were calculated. The average mutations per sequence like transitions and transversions of the PR and RT gene were calculated by the highlighter. The 56 PR and 51 RT gene transitions and transversions value with reference AB023804 were (transitions =10.5 and 32.7) and (transversions = 2.3 and 8.9). The 56 PR and 51 RT gene transitions and transversions value with reference HXB2 were (transitions = 23.5 and 78.7) and (transversions = 10.4 and 21.0).

### Hyper mutation:

Hyper mutated sequences of the genomic DNA were detected through the identification of an excessive G→ A change pattern consistent with APOBEC3G/F signature. Here the 56 PR gene and 51 RT gene sequences were taken for hyper mutation analysis with the reference sequences AB023804 and HXB2. No hyper mutation (P value =1) was observed in the PR and RT gene of HIV-1 subtype C with refence sequences AB023804 and HXB2 (P-value less than 0.05 to indicate a hyper mutant). The diversity of the HIV genome is a major factor due to error-prone reverse transcription, recombination, etc. Diversity trends lead to the failure of the immune system [22]. To see diversity of the HIV-1, the pattern of amino acids of the protease and reverse transcriptase gene has been characterized through the VESPA method, as it is associated with drug resistance towards first-line antiretroviral therapy. The primers of the WHO dried blood spot protocol 2010 was used for analysis of drug resistance mutations in low levels of RNA copies patients (≤40 copies/ml). Thus, this protocol is useful for genotyping studies of archived DNA samples. Patients with drug resistance mutations during low-level viremia were associated with a higher risk of therapy failure. In our study, out of nine patients, two patients had nucleoside reverse transcriptase inhibitor (NRTI), and non-nucleoside reverse transcriptase inhibitor (NNRTI)-associated drug resistance mutations M184V, M184I and Y181C, H221Y and M230I. The two patients were at a higher risk of therapy failure [23]. Characterization of HIV-1 proviral DNA would be helpful in the monitoring of HIV drug resistance variants clinical impact of regimens and treatment success [24]. Although our samples are limited to nine numbers, genotyping tests may be giving valuable information that would be helpful for HIV drug-resistant variants in the region of the Northern part of India. Proviral DNA sequencing provides additional information on drug resistance mutations that impacts on treatment decision and transmission dynamics in a community program [13]. Our findings of the proviral DNA testing of nine long-term ART users (>1 to<7 years) may have some impact on treatment decisions and transmission dynamics. The study of nucleotide substitution is very important to understanding the evolutionary features of the virus and its biological implications [25]. For this reason, our data on the nucleotide substitution ratio (synonymous to non -synonymous) of the PR and RT genes of archived DNA would be important in evolutionary studies. The apo-lipoprotein B mRNA editing catalytic polypeptide-like 3 (APOBEC3 (A3)) protein played an important role in HIV-1 evolution and drug resistance [26]. In our study findings, the nine patients had no G to A mutations. This could be due to the blocking of Vif by the A3 genes in the host. The patients were immunologically tolerable to HIV-1 infection. These nine pro-viral DNAs could be useful in new drug design as all the sequences may be useful in the design of the model to prevent APOBEC3 degradation. When the RT gene and PR gene of the archived DNA were compared with the reference sequences (AB023804 and HXB2), the variation in the genomic signatures were observed. From the observation, we concluded that, genomic signatures are not fixed but changes with time in an evolutionary manner. The evolved genomic signatures may help in the transmission and spread of the virus in many countries. In this North India population, the genomic signature evidence indicates the transmission of non-hypermutants of HIV-1 subtype C in the Agra region, North India.

The HIV-1 has a high mutation rate in the polymerase gene [27]. The genomic variation has been observed in the drug resistance patients through acquired transmission of virus [28]. The high degree of genetic relatedness was observed among the first line ART failure patients over 7 years of first line ART. This study was focused with the existence of genetic variation in the protease and reverse transcriptase gene, the several methods to distinguish the strains, viral epidemiological signatures, nucleotide substitution rates and transmission events by clustering a phylogenetic tree related to one subtype C, hypermutation of analysis of the reverse transcriptase gene associated with APOBEC3 mediated immunity and entropy of the protease and reverse transcriptase gene of drug resistant isolates. This analysis could support the molecular epidemiology investigation of several HIV-1 drug resistant isolates of North Indian population. HIV-1 is known to genetic variation under immune pressure or drug pressure within the human beings [29,30, 31]. HIV-1 is a quasi-species, highly mutate in primary infected patients [32]. The signatures on the HIV-1 DNA polymorphism suggest the reconnection of the present virus of one region with other region of past isolates [33]. From this study, when we compared the protease gene by VESPA analysis with the HIV-1 origin HXB2 isolate and Indian isolates AB023804, 10 amino acid changes position and 4 amino acid changes position were observed. It tempted us to suggest that, the signature pattern of amino acids in the protease gene gradually minimize from the time of origin of virus. When we compared the reverse transcriptase gene by VESPA analysis with the HIV-1 origin HXB2 isolate and Indian isolate AB023804, 17 amino acid changes position and 6 amino acid changes position were observed. From this evidence, we suggest that, there is a genomic drift in the protease and the reverse transcriptase gene during the viral transmission and replication within the human being.

The human apolipoprotein B mRNA editing enzyme catalytic polypeptide like 3G (APOBEC3G or hA3G) belongs to which act as a potent host restriction factor through cytidine deaminase activity [34,35,36]. Human APOBEC3G/F (hA3G/F) confines HIV-1 replication through G-to-A hypermutations that could generate drug-resistant progenies with or without antiretroviral therapy. Human APOBEC3G/F mediated hypermutation is associated with development of drug resistant mutants of HIV-1 subtype C infected Southern Indian patients in first line ART [17]. But in case of Northern Indian patients, even the patients were infected with HIV-1 subtype C drug resistant isolates, no hypermutation (G to A) was observed. Data shows that the North Indian patients were more protected with innate immunity towards the drug resistant isolates. We suggest for the physiological and clinical study of the patients are essential those are living with drug resistant viruses. Signalling complexity of the genome is measured by Shannon entropy and could be helpful in medicine [37]. Thus, there is more genome complexity and hence more entropy. In this study, when we measured the Shannon entropy of the PR and RT gene of drug resistant isolates with the HXB2 and AB023804 isolates, we found there is no change in entropy in the highest measured amino acid positions in both genes. Thus, it can be inferred that, the amino acid complexity remains same from the virus origin with several drug pressure within the human being.

The enormous genetic diversity within the infected individuals with implications for vaccine design and drug treatment was observed. The new infection results the transmission of the virus in a homogeneous viral population in early infection. The diversification of the transmitted virus provides information about the selection pressures during the transmission of the virus within the host [38]. In this study, selection pressure of the amino acids of the drug resistant isolates of the PR and RT gene is more with HXB2 than AB023804. Thus, the biasness amino acid changes in the HIV-1 drug-resistant mutants are a cause of rise of pressure at present context.

## Conclusion:

The analysis of the drug resistance mutation in the protease and reverse transcriptase genes of the archived DNA of HIV-1 subtype-C infected patients over 1 to ≤ years of first-line ART may be helpful in the treatment guideline. A few signature amino acids persisted in the reverse transcriptase and protease genes of archived DNA and the RNA samples from plasma in similar HIV-1 subtype C infected patients over 1 to ≤ years of first-line ART in comparison to the reference sequence. This drug resistance mutation testing could be an important tool in archived DNA, when RNA testing becomes unsuccessful and in vitro DNA is amplified from RNA samples in the plasma of HIV-1 patients.
